# Perceived impact of coronavirus pandemic on uptake of healthcare services in South West Nigeria

**DOI:** 10.11604/pamj.2021.40.26.28279

**Published:** 2021-09-09

**Authors:** Olamide Olajumoke Afolalu, Oluwabusolami Esther Atekoja, Zaccheus Opeyemi Oyewumi, Semiu Opeyemi Adeyeye, Karimat Itunu Jolayemi, Oluwadamilare Akingbade

**Affiliations:** 1 Department of Nursing Science, Osun State University Osogbo, Osun State, Nigeria,; 2 Department of Nursing Science, Olabisi Onabanjo University, Ogun State, Nigeria,; 3 Department of Nursing, Ladoke Akintola University of Technology, Open and Distance Learning Centre, Ogbomoso, Oyo State, Nigeria,; 4 Department of Nursing Science, Adeleke University, Osun State, Nigeria,; 5 The Nethersole School of Nursing, The Chinese University of Hong Kong, Hong Kong, China,; 6 Institute of Nursing Research, Nigeria

**Keywords:** COVID-19, uptake, healthcare systems, Nigeria

## Abstract

**Introduction:**

the COVID-19 pandemic since its emergence has posed a great danger to the health of the general populace while impacting the Nigerian healthcare delivery significantly. Since its emergence, the health system has been stretched with overwhelming responsibilities. The study assessed health providers´ perceived impact of coronavirus pandemic on the uptake of health care services in South West Nigeria.

**Methods:**

a descriptive cross-sectional design using an online structured survey was used to elicit responses from 385 Nigerian health workers selected by convenience sampling technique. Data analysis was done with the Statistical Package for Social Sciences (SPSS) version 26. Comparison of the uptake of healthcare before and during the COVID-19 pandemic was performed using the Chi-square test.

**Results:**

findings revealed a significant difference between the uptake of health care prior and during the COVID-19 pandemic (χ^2^= 92.77, p=0.000) as 253 respondents (65.7%) reported that the hospital recorded a low turn-out of patients during the pandemic and 184 (47.8%) indicated that some of the facility units/departments were temporarily closed due to COVID-19 pandemic. Similarly, there was a significant difference between health-related conditions requiring hospital admission before and during COVID-19 pandemic (χ^2^=3.334 p=0.046). Factors influencing uptake of health services during the COVID-19 pandemic are: fear of nosocomial infection, fear of stigmatization, and misconception/misinformation on COVID-19 diseases and care.

**Conclusion:**

the Nigerian health system in the past months has been remarkably impacted by the pandemic. This calls for immediate restructuring to maintain an equitable distribution of care, while minimizing risk to patients and health providers.

## Introduction

The World Health Organization declared the COVID-19 pandemic a Public Health Emergency of International Concern (PHEIC) on January 30^th^, 2020 [1]. Since the first case was identified in Wuhan, China on December 30^th^ 2019 [2]; the impact of the pandemic on the world has been massive, affecting 219 countries with a total of 102,942,987 confirmed cases and 2,232,233 deaths reported to World Health Organization (WHO) as of 2^nd^ February, 2021 [3]. Since the first case was recorded in Nigeria on February 27^th^, 2020 [4], the country has recorded a total of 133,552 cases, 1,613 deaths and 107,551 recoveries [5] and there are huge concerns that the new COVID-19 variant has been fueling Africa´s second wave of the pandemic [6].

As the virus spreads at alarming rates, the fallout has revealed the global ill-preparedness of governments, health systems and social safety networks to respond to the longstanding and emerging needs of people worldwide. This is not unique to the COVID-19 outbreak; as this was the case in the 2014 Ebola outbreak [7] when the Ebola virus disease (EVD) outbreak in West Africa forced many hospitals to close down or reduce their activity, either to prevent nosocomial transmission or due to staff shortages [8]. Although the 2014-2015 Ebola outbreak was devastating as the increased number of deaths caused by measles, malaria, HIV/AIDS and tuberculosis attributable to health system failure exceeded the deaths from Ebola [8]; however, the impact of COVID-19 pandemic on the health system is worth considering as over 102 million cases recorded across the world with 2,232,233 deaths so far [3] has placed the pandemic as one of the worst infectious disease outbreaks in history [9].

Globally, COVID-19 has also taken its toll on access to sexual and reproductive health care services, which could have serious consequences for women's health, according to the United Nation (UN) population fund [10]. This is the case too in Nigeria. Since the first COVID-19 case was reported in Nigeria, WHO reported that 362,700 women missed ante-natal care in Nigeria between March and August 2020 with 310 maternal deaths in August 2020 nearly double the maternal mortality in August 2019 [11]. Although the Nigerian healthcare system is weak with the country placed as 187^th^out of 195 countries on a global health care index in terms of healthcare delivery [12]; the pandemic has stretched the health system even further [13], culminating in severe decline in services rendered in the hospitals as a survey revealed an acute decline in elective surgeries conducted [14] and antenatal care services provided as several hospitals turned down patients since most do not have facilities to carry out testing and the risk to the staff is high [15]. As isolation centres are constructed, there has been a redeployment of health personnel to combat the virus; thus, making the shortage of manpower obvious in some hospitals, with a resultant implication on patient care.

The toll of the pandemic on health workers has been massive. While the World Health Organization reported that over 10,000 health workers in Africa were infected with the virus in July 2020 [16], the number has increased with Nigeria recording as much as over 1000 deaths in December 2020 [17] and 75 testing positive in one week [18]. Recognizing the fact that the pandemic could strain a health system to the point of rendering it non-functional in providing safer and effective care, the World Health Organization released guidelines to help countries maintain essential health services during the COVID-19 pandemic [19]. The guideline emphasized how countries must identify essential services and prioritize delivery of these services amid the limited resources available to ensure masses are well catered for while the pandemic lasts. However, little has been documented about the uptake of healthcare services in Nigeria amid the pandemic.

In addition to the scarce information available on uptake of healthcare services during the pandemic; the perception of health workers who are at the fore front of providing care for the Nigerian populace amid the pandemic has been under-researched. It is against this background that the researchers set out to assess the perceived impact of COVID-19 on the utilization of healthcare services in South West Nigeria nine weeks after commencement of the lockdown.

**Objectives:** to assess the perception of health workers on the impact of COVID-19 pandemic on the utilization of healthcare services; to investigate the pattern of health care utilization before and during COVID-19 pandemic; to identify the factors influencing uptake of health services during the COVID-19 pandemic; to identify the most common health-related conditions requiring hospitalization during COVID-19 pandemic; to evaluate health care services affected by COVID-19 pandemic.

**Hypotheses:** there is no significant difference between the uptake of health care prior and during the COVID-19 pandemic; there is no significant difference between health-related conditions requiring hospital admission before and during COVID-19 pandemic.

## Methods

**Study design:** the study utilized a descriptive cross-sectional design using an online structured survey.

**Setting:** the study setting was units or departments of public, private, or mission hospitals across the Southwestern part of Nigeria.

**Participants:** participants comprised 385 health care providers (nurses, doctors, pharmacists, laboratory scientists, community health extension workers, radiologists, among others) working in various units/departments of public, private, or mission hospitals across the Southwestern part of Nigeria. All health professionals directly involved in patient care and those in the managerial or administrative fields were included in the study. The exclusion criteria included all students in various professional fields or staff who are not directly involved in patient care and those who did not consent to participate in the study. Due to the prevailing nature of lockdown and the possibility of some members not having access to the internet, a convenience sampling technique was used to select respondents for the study.

**Outcome:** the outcome was the uptake of health care services which refers to utilization of healthcare services by the general populace.

### Data sources

**Instrument for data collection:** the United States national health centre survey tool on COVID-19 and its effect on health centre operations and staff were adapted and modified to fit the study´s objectives [20]. The study utilized an electronic web-based questionnaire designed with the use of Google form. The 29 items structured questionnaires comprising 6 sections with a mix of open and close-ended and Likert types of scale comprised the following: section A: socio-demographic data; section B: impact of coronavirus pandemic on the utilization of health care; section C: pattern of health care utilization before and during coronavirus pandemic; section D: perceived factors influencing the uptake of health services during coronavirus pandemic; section E: common health-related conditions requiring hospitalization during coronavirus pandemic; section F: health care services greatly affected by coronavirus pandemic.

**Instrument validation and reliability:** the face validity and content validity of the modified instrument were determined by the research team. The face and content validity of the instrument was determined in terms of its relevance to the research objectives, length of the questionnaire, layout and format. Suggestions and opinions offered by each researcher served as the basis for the final review of the instrument that was subsequently subjected to a pilot study conducted among 10% of the sample population. These respondents were purposefully selected and not were part of the main study. The data obtained were analysed to ascertain the internal consistency of the instrument and a Cronbach alpha value of 0.74 indicated instrument reliability.

**Study size:** the sample size of 385 health providers from the entire population of health workers in the South Western part of Nigeria was determined using Cochran´s formula for yielding a representative sample for proportions in large populations [21]:

$$n_{0}=\frac{z^{2}\times p\times q}{e^{2}}$$

Where: n=sample size; P= (estimated) proportion of health providers who perceived coronavirus pandemic has an impact on uptake of healthcare services in the previous studies = 50% or 0.5; q=1-p =0.5; Z=A 95% confidence level and at least ± 5% precision will give a standard normal Z value of 1.96; e=desired level of precision (i.e. the margin of error) = 0.05;

$$\begin{array}{l} n=\frac{z^{2}\times p\times q}{e^{2}}\\ \frac{\left(1.96^{2}\right)\times 0.5\times 0.5}{{\left(0.05\right)}^{2}}=\frac{\left(1.92^{2}\right)\times 0.25}{0.025}=384.16 \end{array}$$

### Statistical methods

**Methods of data collection:** prior to commencement of data collection, a one-page recruitment poster was designed. This was made available to various associations of health professionals in South West Nigeria who helped disseminated the survey to their various organizational social media platforms. Similarly, the survey was posted on various social media pages (WhatsApp, Instagram, Messenger, Facebook and twitter) to sensitize health professionals on the survey. Care was taken to cover social media platforms across all the six south-western States to ensure representative sampling across the States. Some of the participants were also invited via text messages and through their emails. The poster provided information about the study title, aims and procedures. The combination of these methods helps to maximize the content validity and minimize coverage error [22]. Recruitment of prospective participants was voluntary without any incentive or compensation. Informed consent was sought from each participant by completing a declaration page embedded in the online questionnaire with a ‘YES´ or ‘NO´ response. Those who indicated a willingness to participate by giving an affirmative response to the declaration statement had free access to complete the questionnaire, while those who gave a “NO” response were denied access to the questionnaire page. The survey was completed within 7-10 minutes. Data collection was undertaken between 18^th^ to 31^st^ July 2020.

**Method of data analysis:** data analysis was conducted using descriptive and inferential statistics. Continuous variables were computed using mean and standard deviations while categorical data were computed using frequency and percentages. Comparison of the uptake of healthcare before and during the COVID-19 pandemic was performed using the Chi-square test. A statistical significance level of 0.05 was assigned for all statistical analyses. Statistical Package for Social Sciences (SPSS) software 26 was used for the data analysis.

**Ethical consideration:** written approval was obtained from the Ethics and Research Committee of the University of Medical Science (UNIMED), Ondo and Ladoke Akintola University of Technology (LAUTECH), Osogbo (protocol number: LTH/EC/2020/08/470). The researchers were guided by the principles for ethical research as stated in the Declaration of Helsinki (General Assembly of the World Medical Association, 2014).

## Results

As shown in Table 1, the socio-demographic status revealed that most respondents 275 (71.4%) are within the age range 21-40 years. A significant number are females 243 (63.1%), who are married 263 (68.3%) and are Christian 264 (68.6%). A significant proportion are nurses 184 (47.8), who hold a bachelor´s degree 227 (59.0%). Many 134 (34.8%) have 11-20 years of practice experience and work in urban settings 211 (54.8%), in government type of facilities 303 (78.7%) and in secondary level of healthcare 150 (39.0%). Majority of the respondents had their work facilities located in Lagos 83 (21.6%).

**Table 1:** socio-demographic characteristics (n=385)

Variables	n (%)
**Age (years)**	
Less than 20	3 (0.8)
21-40	275 (71.4)
41-60	107 (27.8)
**Gender**	
Female	243 (63.1)
Male	142 (36.9)
**Marital status**	
Divorced/separated	6 (1.6)
Married	263 (68.3)
Single	103 (26.7)
Widow/widower	13 (3.4)
**Religion**	
Christianity	264 (68.6)
Islam	118 (30.6)
Traditional	3 (0.8)
**Healthcare cadre/profession**	
CHEW	28 (7.3)
CHO	10 (2.6)
Medical doctor	60 (15.6)
Medical laboratory scientist	31 (8.1)
Medical laboratory technician	7 (1.8)
Nursing	184 (47.8)
Nutritionist	12 (3.1)
Others (specify)	9 (2.3)
Pharmacist	28 (7.3)
Pharmacy technician	5 (1.3)
Radiotherapist	11 (2.8)
**What is your highest qualification?**	
Bachelor's degree	227 (59.0)
Diploma	87 (22.6)
Masters	65 (16.9)
PhD	6 (1.55)
**Years of practice**	
Less than 10 years	115 (29.9)
11-20 years	134 (34.8)
21-30 years	69 (17.9)
31-40 years	67 (17.4)
**In what setting is your place of employment?**	
Rural (outside the city)	63 (16.4)
Suburban (residential area bordering a city)	111 (28.8)
Urban (in the city)	211 (54.8)
Type of facility	
Government	303 (78.7)
Mission	13 (3.4)
Private	69 (17.9)
**If a government facility, please specify the level of the facility**	
Not applicable	26 (6.7)
Primary	75 (19.5)
Secondary	150 (39.0)
Tertiary	134 (34.8)
**What state is your facility located?**	
Lagos	83 (21.6)
Ekiti	72 (18.7)
Ogun	66 (17.1)
Ondo	51 (13.2)
Osun	53 (13.8)
Oyo	60 (15.6)

CHEW: community health extension worker; CHO: chief happiness officer

**Perceived impact of COVID-19 pandemic on Nigeria healthcare services (n=385):** findings show a significant impact of COVID-19 pandemic on Nigeria healthcare services; 184 (47.8%) indicated that some of the facility units/departments were temporarily closed due to COVID-19 pandemic; out of which, majority 140 (76.1%) indicated 1-4, and 44 (23.9%) reveals that 5-8 facilities were temporarily closed. Majority 326 (84.7%) revealed that none of their staff had tested positive to COVID-19, while 59 (15.3%) indicated that more than one of their staff had tested positive to the virus. Additionally, 366 (95.1%) revealed that up to 0-7 of their staff members have been temporarily transferred to isolation centres, while 19 (4.9%) indicated the transfer of 8-15 staff. Majority of the respondents 232 (60.3) showed that none of their staff could commence compulsory annual leave due to COVID-19, while 153 (39.7%) revealed commencement of leave among 1-20 staff. A significant number of respondents 346 (89.9%) confirmed that COVID-19 pandemic has an impact on patient utilization of medical services, while 39 (10.1%) have an opposing view (Table 2).

**Table 2:** impact of COVID-19 pandemic on Nigeria healthcare services (n=385)

Variables	n (%)
**Has any of your facility units/departments been temporarily closed due to COVID-19?**	
Yes	184 (47.8)
No	201 (52.2)
**If yes, how many?**	
1-4	140 (76.1)
5-8	44 (23.9)
Total	184 (100.0)
**How many staff members at your health center have tested positive for COVID-19?**	
1-5	34 (8.8)
Greater than 5	25 (6.5)
None	326 (84.7)
**How many staff members in your health facility have been temporarily transferred to isolation centers due to COVID-19?**	
0-7	366 (95.1)
8-15	19 (4.9)
**How many staff have commenced compulsory annual leave due to COVID-19?**	
None	232 (60.3)
1-20	153 (39.7)
**Do you think COVID-19 pandemic has any impact on patient utilization of medical services**	
Yes	346 (89.9)
No	39 (10.1)

**The pattern of healthcare utilization prior and during COVID-19 Pandemic (n=385):** as shown in Table 3, the pattern of healthcare utilization prior and during the COVID-19 pandemic reveals that 188 (48.8%) health workers often attend to less than 25 patients in the day before the pandemic, compared to 236 (61.3%) during the pandemic. Moreover, 136 (35.3%) compared to 110 (28.6%) reportedly saw 26-50 patients before COVID-19, while 26 (6.8%) respondents attended to 76-100 patients before the pandemic compared to 6 (1.6%) during the pandemic.

**Table 3:** pattern of healthcare utilization prior and during COVID-19 pandemic

No of patients attended to	Prior n (%) mean score=32.7	During n (%) mean score=24.9	X^2^ (P-value)
0-25	188 (48.8)	236 (61.3)	92.77 (0.000)
26-50	136 (35.3)	110 (28.6)	
51-75	32 (8.3)	32 (8.3)	
76-100	26 (6.8)	6 (1.6)	
Above 100	3 (0.8)	1 (0.3)	

**Figure 1:**
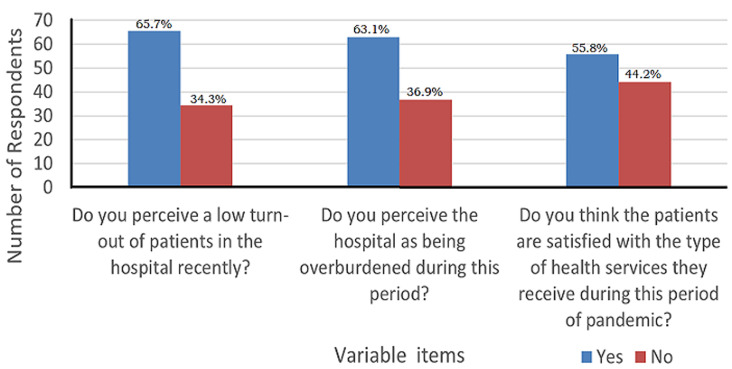
pattern of healthcare utilization during COVID-19 pandemic

Moreover, a significant number of respondents, 253 (65.7%) reported that the hospital recorded a low turn-out of patients during the COVID-19 pandemic, compared to 243 (63.1%) who indicated that the hospitals were not overburdened during the pandemic (Figure 1). Majority 148 (38.4%) perceived the patient´s preference for home care during the pandemic compared to 96 (25.0%), who said patients will prefer hospital. It was also revealed that a good number of respondents 170 (44.2%) felt that patients are not satisfied with the type of health services received during the period of pandemic. The Chi-square test analysis (92.77 (1), p=0.000) revealed that there is a significant difference between the uptake of health care prior to and during COVID-19 pandemic (Table 3).

**Factors influencing uptake of health service during COVID-19 pandemic (n=385):** several factors were established to affect the uptake of healthcare services during the COVID-19 pandemic (Table 4). A significant number of respondents, 287 (74.5%) affirmed that fear of nosocomial infection affects health services rendered during the pandemic. Other factors identified include lack of available infection control and prevention services 244 (63.4%), stigmatization 284 (73.2%), adverse health outcomes resulting from accessibility barriers 248 (64.4%) and the attitude of healthcare providers/nature of the hospital atmosphere during pandemic 224 (58.2%) and misconception/misinformation on COVID-19 diseases and care 286 (74.3%). Table 5 reveals that a significant proportion of the respondents perceived patients with chronic disease 274 (71.2%) and individuals requiring surgical procedures 297 (77.2%) as the most common health-related conditions requiring hospitalization during the COVID-19 pandemic.

**Table 4:** factors influencing uptake of health service during COVID-19 pandemic

Variables	Agree	Neutral	Disagree
Fear of nosocomial infection	287 (74.5%)	48 (12.5%)	50 (13.0%)
Lack of available infection control and prevention services	244 (63.4%)	67 (17.4%)	74 (19.2%)
Fear of stigmatization	284 (73.2%)	46 (11.9%)	57 (14.8%)
Adverse health outcomes resulting from accessibility barriers	248 (64.4%)	82 (21.3%)	55 (14.3%)
Attitudes of healthcare providers/nature of hospital atmosphere during pandemic	224 (58.2%)	74 (19.2%)	87 (22.6%)
Misconception/misinformation on COVID-19 diseases and care	286 (74.3%)	54 (14.0%)	45 (11.7%)
Information on facility management	240 (62.3%)	54 (14.0%)	91 (23.6%)
Long waiting times	219 (56.9%)	91 (23.6%)	75 (19.5%)
Lack of available trained personnel	184 (47.8%)	54 (14.0%)	147 (38.2%)
Cost of drugs and treatment	244 (63.4%)	66(17.1%)	75 (19.5%)

**Table 5:** health-related conditions requiring hospitalization during COVID-19 pandemic

Variables	n=423 (%)	Mean	Standard dev.
**Patient with chronic diseases such as cancers**			
Extremely unlikely	59 (15.3%)	3.59	1.404
Unlikely	35 (9.1%)		
Neutral	17 (4.4%)		
Likely	159 (41.3%)		
Extremely likely	115 (29.9%)		
**Individuals requiring surgical procedures**			
Extremely unlikely	37 (9.6%)	3.88	1.300
Unlikely	32 (8.3%)		
Neutral	19 (4.9%)		
Likely	157 (40.8%)		
Extremely likely	140 (36.4%)		
**Individuals with non-communicable diseases like hypertension, diabetes, etc**			
Extremely unlikely	87 (22.6%)	3.16	1.499
Unlikely	62 (16.1%)		
Neutral	40 (10.4%)		
Likely	126 (32.7%)		
Extremely likely	70 (18.2%)		
**Individual with mental disorders and psychiatric conditions**			
Extremely unlikely	48 (12.5%)	3.47	1.329
Unlikely	56 (14.5%)		
Neutral	57 (14.8%)		
Likely	148 (38.4%)		
Extremely likely	76 (19.7%)		
**Drug/substance abuse-related rehabilitation patients**			
Extremely unlikely	53 (13.8%)	3.31	1.37
Unlikely	83 (21.6%)		
Neutral	50 (13.0%)		
Likely	133 (34.5%)		
Extremely likely	66 (17.1%)		
**Dental problems and surgeries**			
Extremely unlikely	88 (22.9%)	3.18	1.486
Unlikely	61 (15.8%)		
Neutral	36 (9.4%)		
Likely	135 (35.1%)		
Extremely likely	65 (16.9%)		
**Individuals with health conditions such as malaria, typhoid, headache, stress syndrome**			
Extremely unlikely	85 (22.1%)	3.25	1.493
Unlikely	50 (13.0%)		
Neutral	32 (8.3%)		
Likely	143 (37.1%)		
Extremely likely	75 (19.5%)		

**Findings on evaluation of health care services affected by coronavirus pandemic:** the results revealed that regular antenatal services 152 (39.5%), immunization and family planning services 160 (41.6) and emergency services 154 (40.0%), were the least affected by the pandemic; while in-patient and out-patient services 186 (48.3%), emergency services 153 (39.7%) and surgery and medical services 159 (41.3%) are usually affected. Laboratory and pharmaceutical services are rarely affected by pandemic 131 (34.0%) (Table 6). The Chi-square test analysis (3.334 (1), p=0.046) revealed that there is a significant difference between health-related conditions requiring hospital admission before and during COVID-19 pandemic.

**Table 6:** evaluation of health care services affected by coronavirus pandemic

Variable	Never	Rarely	Usually
Antenatal services, obstetrics and gynaecologic services	152 (39.5%)	93 (24.2%)	140 (36.4%)
In-patient and out-patient services	93 (24.2%)	106 (27.5%)	186 (48.3%)
Emergency services	154 (40.0%)	78 (20.3%)	153 (39.7%)
Laboratory services and pharmaceutical services	137 (35.6%)	117 (30.4%)	131 (34.0%)
Surgery and medical services	115 (29.9%)	111 (28.8%)	159 (41.3%)
Immunization services and family planning services	160 (41.6)	84 (21.8)	141 (36.6)

## Discussion

This interprofessional-based study provides a snapshot of the perceived impact of coronavirus on the uptake of healthcare services in Nigeria, after nine (9) weeks of movement restriction and lockdown. To the best of our knowledge, this study was among one of the first to investigate the immediate impact of COVID-19 on the utilization of health care services among Nigerian healthcare workers. The web survey was concluded on 31^st^ July 2020. The study reveals that the cadre of health workers across the professions fall within the younger age group of 21-40 years, which could be linked to the younger population at lesser risk of contracting the virus than the older age groups among whom over 95% cases of coronavirus death occurred [23,24]. Similarly, the study showed that only 59 (15.3%) indicated that more than one of their staff had tested positive to the virus. However, more recent statistics revealed that there were 1000 deaths of health workers in December 2020 and as much as 75 testing positive in one week in January 2021 [14,15]. This is alarming and more efforts must be geared towards safeguarding the health of the health workers battling the pandemic.

Majority of the respondents are females 243 (63.1%). A significant proportion were nurses 184 (47.8), followed by medical doctors 60 (11.2%); with the majority holding a bachelor´s degree 227 (59.0%). The wide variation in the number of these health professionals can be buttressed with the observatory study carried out in Nigeria which states that there are about 35 doctors and 86 nurses per 100,000 population [25]. This can also be compared to a sub-Saharan average of 15 doctors and 72 nurses per 100,000 population [26]. The territorial coverage of our online survey extends to all 6 States in South West Nigeria.

Findings revealing the impact of the COVID-19 pandemic on healthcare services reflect that the pandemic has dramatically changed how outpatient care is delivered in health care practices. This study shows that 346 (89.9%) health care workers confirmed that COVID-19 pandemic influenced patient utilization of medical services, which was buttressed by 184 (43.5%) respondents confirming temporary closure of some facilities; and subsequent temporary redeployment of 0-7 staff members to isolation centres (366, 95.1%). Furthermore, majority 232 (60.3%) confirmed that none of their staff members went on compulsory annual leave as a result of the pandemic. These findings revealed the various ways COVID-19 has changed the healthcare services rendered to the patient and the ill-preparedness of governments, hospital agencies and social safety networks that provide long-standing and emergency care to sick people in times of crisis [25]. Several other studies have revealed other dramatic changes COVID-19 has on healthcare practices where there were numerous cancellations of hospital appointments and postponement of surgeries [27,28].

Results from the study revealed that the rate at which people seek healthcare services during the COVID-19 pandemic has reduced when compared to the pattern prior the pandemic. Statistics showed that 136 (35.3%) compared to 110 (28.6%) respondents reportedly saw 26-50 patients before COVID-19 and 26 (6.8%) respondents attended to 76-100 patients before the pandemic compared to 6 (1.6%) during the pandemic. This shows that patients visit the hospital less during the pandemic. This finding is similar to a study carried out in Korea during the outbreak of Middle East respiratory syndrome (MERS), which reported 12.2% and 13.4% decrease in the total number of patients who visited the emergency department (ED) of a Korean hospital during the MERS-CoV epidemic period of 2014 and 2013 respectively [29]. Additionally, a study conducted in Sierra Leone also revealed that during the Ebola virus disease outbreak the number of in-hospital deliveries and C-sections decreased by over 20% [8].

The three most identified factors influencing the uptake of health services during the COVID-19 pandemic are: fear of nosocomial infection 287 (74.5%); fear of stigmatization 284 (73.2%); misconception/misinformation on COVID-19 diseases and care 286 (74.3%). These findings can be compared to a West African study where many hospitals were forced to close or reduce their activity, for the fear of contracting hospital-acquired infections [8]. Meanwhile, health workers have been leveraging on telehealth to keep their organizations running, although telehealth volumes have not been enough to completely offset the drop in in-person visits [30]. It is a great alternative to help reduce the number of people who visit health facilities. The apparent potential of telemedicine at the global level shows that it could assist developed and underdeveloped countries to manage the increasing number of COVID-19 cases [31]. Nigeria can greatly benefit from the telehealth boom to minimize staff exposure to ill persons and preserve personal protective equipment (PPE) even as statistics revealed that the mobile internet usage in Nigeria grew from 17.2 million in 2015 to 85.26 million in 2020 and is expected to grow to 151.3 million by the end of 2025 [32].

The fears of stigmatization identified in this study has been reported in a study that reveals feelings of guilt and stigma as often associated with prolonged quarantine; this could be aggravated by an already existing psychological factor [33], including posttraumatic stress symptoms, confusion and anger [34]; their negative effects could be reduced if well planned.

A significant proportion of the respondents perceived patients with chronic disease 274 (71.2%) and individuals requiring surgical procedures 297 (77.2%) as the most common health-related conditions requiring hospitalization during the COVID-19 pandemic. This finding corroborates a report that described chronic disease as a potential burden to population health, having an impact on life expectancy, health systems and finances [35]. Even though, running regular out-patient clinics for non communicable diseases (NCDs) can be a little challenging due to contact with those presenting with flu-like symptoms or suspected cases of COVID-19; alternative strategies are needed to be implemented. This will serve as a measure for promoting beneficence while reducing the risk of violating non-maleficence [19]. The decline in surgical procedures amid the pandemic was also reported by a recent study [14]. This calls for concerted efforts to meet patients with surgical needs amid the pandemic.

Healthcare service disruptions reported at the global level call for immediate changes in the modalities of care to urgently avert crises associated with inadequate access to health care services, as some health conditions require periodic review of medications, therapy and laboratory investigations, among others. Our results reveal that in-patient and out-patient services 186 (48.3%), emergency services 153 (39.7%) and surgery and medical services 159 (41.3%) are mostly affected.

The main finding from this study is that health services have been partially or completely disrupted in many countries. The WHO report of countries surveyed during COVID-19 pandemic reveals that about 53% of the countries surveyed have partially or completely disrupted services for hypertension treatment; treatment of diabetes and its related complications (49%); cancer treatment (42%) and cardiovascular emergencies (31%); which are important health conditions mostly receiving care from in and/or out-patient, as well as emergency departments [36]. Contact with the health care system for accessible treatment is imperative, more importantly for people who use drugs and in need of regular review to reduce the risk of substance use-related problems and promote effective communication of health messages during the pandemic [37].

**Strength and limitations:** the uptake of healthcare services in South West Nigeria during the COVID-19 pandemic presented with the STROBE (Strengthening the Reporting of Observational Studies in Epidemiology) guidelines for reporting observational studies [38] in this study gave an insightful picture of the early impact of the pandemic on health care utilization during the lockdown from the perspective of healthcare personnel. However, a nationwide study will give a clearer picture of the national situation. Similarly, only health workers with internet access were part of the study. Additionally, the impact of COVID-19 on health care services was based on self-reported perceptions which are subject to social desirability response bias. Hence, the interpretation of the results should be done with caution.

## Conclusion

The health systems in the past months have witnessed a remarkable impact posed by the COVID-19 pandemic. As the situation unfolds, there is a need for restructuring of the health system in a bid to maintain an equitable distribution of care while minimizing risk to patients and health personnel. All hands must be on deck to ensure essential healthcare services are undisrupted while the pandemic lasts. If this is done, the untoward effect of the pandemic on the uptake of healthcare services will be mitigated as efforts are being geared towards flattening the curve.

### What is known about this topic

- The COVID-19 has impacted virtually all dimensions of human existence;
- The health sector is one of the areas most affected by the pandemic.

### What this study adds

- The study provides a clear picture of the early impact of the pandemic on the uptake of healthcare services in Nigeria from the perspective of the health workers;
- The factors influencing uptake of health services during the COVID-19 pandemic were also brought to fore in this study.
